# Case Report: Retrospective discovery of *Theileria orientalis* Ikeda in *Haemaphysalis longicornis* Neumann ticks on a cow-calf farm in Tennessee (US)

**DOI:** 10.3389/fvets.2026.1770304

**Published:** 2026-03-05

**Authors:** Rebecca A. Butler, Lisa I. Muller, Karen C. Poh, Mitzi Aguilar, Kyra S. Hokkanen-Harmon, Jennifer G. Chandler, Daniel Grove, Rebecca T. Trout Fryxell

**Affiliations:** 1 Department of Entomology and Plant Pathology, University of Tennessee, Knoxville, TN, United States; 2 School of Natural Resources, University of Tennessee, Knoxville, TN, United States; 3 Animal Disease Research Unit, Agricultural Research Service, United States Department of Agriculture, Pullman, WA, United States; 4 Department of Biomedical and Diagnostic Sciences, College of Veterinary Medicine, University of Tennessee, Knoxville, TN, United States

**Keywords:** cattle, *Haemaphysalis longicornis* Neumann, *Theileria orientalis* Ikeda, tick, vector-borne disease, wildlife

## Abstract

*Theileria orientalis* Ikeda is a protozoan parasite that has recently been detected in *Haemaphysalis longicornis* Neumann ticks and bovine serum samples in the United States (US). This parasite is known to cause theileriosis in bovine hosts and has negatively impacted the cattle industry worldwide. The transmission of this pathogen at the livestock–wildlife interface in the US is not fully understood. *Theileria orientalis* Ikeda was reported by producers on a cow-calf farm in eastern Tennessee. A retrospective analysis of field- and host-collected *H. longicornis* resulted in the detection of *T. orientalis* Ikeda in nymphal *H. longicornis* ticks collected from cattle (*Bos taurus*), domestic cats (*Felis catus*), raccoons (*Procyon lotor*), and Virginia opossums (*Didelphis virginiana*). Notably, the protozoan DNA was not found in the blood of these hosts. Multiple hosts (cattle, raccoons, Virginia opossums, and a domestic cat) contributed to the presence of *T. orientalis* Ikeda-infected *H. longicornis* ticks on the farm. Treating these hosts with acaricides could be important for reducing tick abundance and pathogen transmission. Additionally, biosecurity practices, such as changing gloves and syringes between handling bovine hosts, are important for preventing the accidental mechanical transmission of *Theileria* parasites.

## Background

The first case of the exotic bovine pathogen *Theileria orientalis* Ikeda genotype in the United States was detected in 2019 in cattle (*Bos taurus*) in Virginia (1) and was later confirmed in longhorned ticks (*Haemaphysalis longicornis* Neumann) collected from the same cattle farm (2). Since its initial detection, *Theileria orientalis* Ikeda has now been reported in the Northeast, Southeast, and Midwest (3). The known vector, *H. longicornis*, has a generalist feeding strategy and has been found infesting a variety of mammalian and avian hosts (4–7). Notably, in other countries where both the vector and pathogen are established, wildlife has been implicated as potential reservoir hosts in the disease cycle or maintenance of the *T. orientalis* Ikeda genotype (8).

As part of a collaborative study attempting to detect and collect *H. longicornis* in Tennessee, we established a passive surveillance network to collect ticks from companion and livestock animals across the state (9). Briefly, ticks were collected from cattle when they were restrained for routine annual vaccinations (IACUC # 2192–0419). During these collections, we detected *H. longicornis* on several farms in the eastern part of the state and attempted to help producers manage their tick populations by providing them with different control options that included on- and off-animal recommendations (10). At these farms, we found that wildlife also supported *H. longicornis* populations, identifying specific wildlife species that serve as important hosts for immature longhorned tick populations, including raccoons (*Procyon lotor*), Virginia opossums (*Didelphis virginiana*), and eastern chipmunks (*Tamias striatus*) (7).

In April 2023, we received a call from the owner of a cow–calf farm participating in our research program. The owner reported that their cattle were presenting with lethargy and general malaise, which were similar to clinical signs associated with theileriosis (1). The owner of the farm described that they have an open herd of approximately 35 cattle (consisting of Limousin, Charolais, Watusi, and Red and Aberdeen Angus breeds) and that they reuse syringes during annual spring vaccinations. Tick management on the farm involved annual mowing (bush hogging) of pastures in the autumn and on-animal application of both GardStar 40% EC Permethrin Concentrate spray (EPA Reg. No. 39039–8) and TriZap insecticide cattle ear tags containing pyrethroids and piperonyl butoxide (PBO) in the spring (Y-Tex Corporation, Cody, Wyoming).

**Figure 1:**
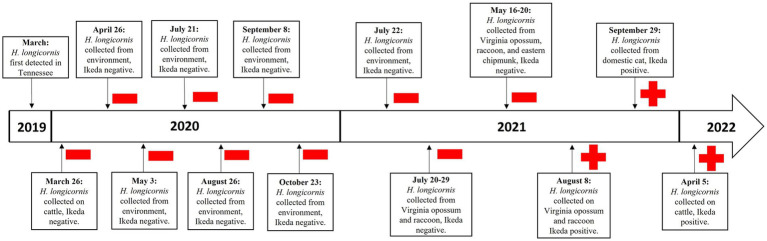
Timeline represents the introduction of *Theileria orientalis* Ikeda in *Haemaphysalis longicornis* ticks collected from the environment and from mammalian hosts, such as cattle (*Bos taurus*), domestic cats (*Felis catus*), raccoons (*Procyon lotor*), Virginia opossums (*Didelphis virginiana*), and eastern chipmunks (*Tamias striatus*), on a farm in eastern Tennessee, 2020–2022.

Recognizing that wildlife can host *H. longicornis* ticks (4, 5, 7), we conducted a retrospective study on *T. orientalis* in tick species from this previously described cow-calf farm that had recorded cases of *T. orientalis* Ikeda-positive animals (Figure 1). Thus, the purpose of this study was to confirm the presence of *T. orientalis* Ikeda in the farm’s tick population and identify tick-host associations with the parasite. To achieve this, we conducted a retrospective analysis of the ticks collected from this farm and screened them for the parasite using polymerase chain reaction (PCR) techniques.

## Methods

### Tick collection

We collected ticks from cattle, wildlife, and the environment at the case site in Union County, Tennessee, for 26 months (Figure 1). Cattle were examined annually in the spring of 2020 and 2022; however, we could not conduct an examination in 2021 due to concerns associated with the SARS-CoV-2 pandemic (11). We trapped small- and medium-sized wildlife hosts each season (winter 2020 to fall 2021) and checked these captured animals for ticks for an entire year (IACUC #2774–0620) (7). The wildlife was handled and identified as previously described, and blood samples were collected from anesthetized animals and stored in tubes containing EDTA at −80^o^ C (12, 13). Beginning in April 2020, ticks were collected at eight different time points from the environment with corduroy drag cloths along nine transects that represented forest, edge, and open-field habitats. A summary of these findings can be found in earlier reports (10). All collected ticks were placed in vials containing 80% ethanol, with each vial labeled according to the specific date and the origin of the samples (environment or host). We identified the ticks by species and life stage under a microscope using taxonomic keys (14–17).

### *Theileria orientalis* Ikeda detection

To confirm *T. orientalis* Ikeda in *H. longicornis,* individual nymph and adult specimens were bisected with a sterile blade such that one half was saved as a voucher specimen and the other half was used for *T. orientalis* Ikeda detection. Due to the small size of the larvae and their lower likelihood of testing positive for *T. orientalis* Ikeda, the larvae were pooled in groups of 10 and screened as single pools. To extract total DNA from each tick, we used the QIAamp 96 DNA kit (Qiagen, Hilden, Germany) in a Qiagen 96-well QIAcube HT (Qiagen, Hilden, Germany). This process yielded 200 μL of total DNA (tick, pathogen, potential host, etc.) eluted in AE buffer (18). We then used 2 μL of the eluted DNA to determine whether the tick (or pool) was *T. orientalis* Ikeda-positive by attempting to amplify the major piroplasm surface protein (MPSP) gene found in *T. orientalis* using quantitative PCR (qPCR) (2). The reactions consisted of 7.5 μL of TaqMan Universal Master Mix 2.0 (Thermo Fisher Scientific, Waltham, MA), 0.45 μL (0.3 uM) of forward (5′-GCA AAC AAG GAT TTG CAC GC-3′) and (0.3 uM) reverse primers (5′-TGT GAG ACT CAA TGC GCC TAG A-3′), 0.2 μL (0.1 uM) of universal probe (5′-NED–TCG ACA AGT TCT CAC CAC-MGB-NFQ-3′), 2 μL of DNA, and 4.45 μL of nuclease-free water (totaling 15 μL per reaction) (1). The amplification conditions consisted of an initial step of 10 min at 95 °C, followed by 45 cycles of 15 s at 95 °C and 1 min at 60 °C (2). Samples were considered *T. orientalis* Ikeda*-*positive if Ct values were 40 or less and rerun using nested-PCR (nPCR) amplifying the MPSP gene (1).

The first reaction consisted of 5 μL of DreamTaq Hot Start Green PCR Master Mix 2X (Thermo Fisher Scientific, Waltham, MA), 0.5 μM each of the forward (5′-CTTTGCCTAGGATACTTCCT-3′) and reverse primers (5′-ACGGCAAGTGGTGAGAACT-3′), 3 μL of eluted tick DNA, and 1 μL of nuclease-free water (totaling 10 μL per reaction). The second reaction consisted of 12.5 μL of DreamTaq Hot Start Green PCR Master Mix 2X, 0.4 μM of each primer, 8.5 μL of water, and 2 μL of DNA from the first reaction. The second reaction products were visualized on a 1.5% agarose gel stained with ethidium bromide, run at 100 V for 1.5 h. Amplicons of ~800–900 bp were considered *T. orientalis* Ikeda positive. To confirm *T. orientalis* Ikeda, the second reaction products were cleaned with ExoSAP-IT (Thermo Fisher Scientific, Waltham, MA) and sent to Eurofins Genomics (Louisville, Kentucky, United States) for bidirectional Sanger sequencing. Sequences were then aligned in BioEdit, a consensus sequence was created, and those consensus sequences were used as a query in the NCBI Basic Local Alignment Search Tool, BLAST. Sequences that were greater than 95% genetically similar to other *T. orientalis* Ikeda sequences in GenBank were considered positive. Nuclease-free water and PCR master mix were used as negative controls, and previously sequenced *H. longicornis,* which was DNA positive for *T. orientalis,* was used as a positive control for all assays.

DNA was extracted from wildlife host blood samples using the Qiagen DNeasy Blood and Tissue kit (Qiagen, Hilden, Germany) following a modified version of the manufacturer’s instructions. Blood samples were prepped and lysed with 100 μL of buffer AL and 2 mg/mL of proteinase K and then vortexed. After incubating at 56 °C for 10 min, DNA was precipitated with 200 μL of 100% ethanol, and the supernatant was transferred to the DNeasy spin column. Sample columns were spun down at 8000 rpm for 1 min, and flow-through was discarded. The column was then washed with 500 μL of buffer AW1, centrifuged for 1 min, and the flow-through was discarded. After a second wash with 500 μL of buffer AW2, the column was centrifuged at 12,000 rpm for 1 min to dry the column. Purified DNA was eluted with 100 μL of water.

To test for *T. orientalis*, the MPSP gene was amplified by nPCR. Each 20 μL reaction consisted of 0.5 μM primers, 10 μL of JumpStart Taq ReadyMix (Millipore Sigma, Burlington, MA), 2 μL of RediLoad Loading Buffer (Invitrogen, Waltham, MA), and nuclease-free water. For external amplification, 1 μL of purified DNA was used, and 1 μL of amplified DNA was used in the subsequent nPCR. The following primers were used for the initial amplification of a 710 bp fragment: MPSP external forward 5′-CAACCAATGCCAACGACGTC-3′ and MPSP external reverse 5′-TGAGACTCAGTGCGCCTAGA-3′. Nested amplification was performed using MPSP internal forward primer 5′-GGCCAAATACACTGCAGTCA-3′ and MPSP internal reverse primer 5′-AACGGCAAGTGGTGAGAACT-3′ for a 232 bp fragment (Janaina Peixoto, unpublished primers). Primers were designed using PCR conditions that are the same for both reactions. After an initial denaturation at 95 °C for 5 min, 35 cycles consisted of denaturation at 95 °C for 30 s, annealing at 60 °C for 30 s, extension at 72 °C for 30 s, followed by an additional 7 min at 72 °C. PCR amplicons were analyzed on a 1% agarose gel stained with SYBR Safe (Invitrogen).

## Results

In March 2020, we collected the first *H. longicornis* ticks from the farm, including 23 females and 3 nymphs from 11 cattle, which presented as healthy animals. In April 2022, 13 cattle were checked for ticks, which had a total of 46 *H. longicornis* (39 nymphs and 7 females). Because the most searched cattle were calves, which were sold after weaning, many could not be checked for ticks each season. We screened these ticks after being notified that cattle presented with clinical signs, and of these 46 specimens, 15 nymphs were qPCR positive for *T. orientalis*. Analysis of the 93 environmentally collected *H. longicornis* (21 larvae, 68 nymphs, and 4 females) from drag cloths in 2020–2022 did not lead to additional qPCR-positive ticks. Analysis of the 178 *H. longicornis* collected from wildlife, specifically 8 Virginia opossums, 16 raccoons, and 1 eastern chipmunk, led to 4 additional *T. orientalis* Ikeda detections, including 3 nymphs collected from a raccoon and 1 nymph collected from a Virginia opossum. One nymph collected from one cat was also qPCR positive. A total of 20 ticks, collected from cattle, wildlife, and a companion animal, were qPCR positive. Detection information by host and month are presented in Table 1. Sequencing results confirmed *T. orientalis* Ikeda in 19 of the 20 qPCR-positive samples. Three *Theileria*-positive *H. longicornis* nymphal ticks, collected from raccoons, were randomly selected for sequencing. All *Theileria* sequences selected were identical, and the sequence obtained from this study was deposited in GenBank under accession number PX933207. The resulting sequences were at least 98% genetically similar to GenBank-submitted sequences from around the world, including the US, Japan, Australia, and the Philippines (AB759043, OR570618, LC684836, JN252703, MG758109, and AB520949). One tick sample, collected from a cow, did not amplify properly and was deemed inconclusive. The corresponding wildlife blood samples were also screened, and all were nPCR negative for *T. orientalis*. Unfortunately, we could not collect blood samples from companion animals or livestock on the farm, at the request of the farm owner.

**Table 1:** Total number of *Haemaphysalis longicornis* ticks screened from the environment and collected from mammalian hosts on a farm positive for *Theileria orientalis* Ikeda in eastern Tennessee from 2020 to 2022.

Year	Month	Sampling origin	Total number of *Haemaphysalis longicornis Theileria* positive/total ticks collected (percent positive)			
			Larvae	Nymphs	Females	Total
2020	March	Cattle (*n* = 11)	—	0/3 (0.00%)	0/23 (0.00%)	0/26 (0.00%)
	April	Environment	0/1 (0.00%)	0/10 (0.00%)	—	0/11 (0.00%)
	May		0/20 (0.00%)	0/28 (0.00%)	0/3 (0.00%)	0/51 (0.00%)
	July		—	0/2 (0.00%)	—	0/2 (0.00%)
	August		—	0/10 (0.00%)	—	0/10 (0.00%)
	September		—	0/8 (0.00%)	—	0/8 (0.00%)
	October		—	0/4 (0.00%)	—	0/4 (0.00%)
2021	May	Virginia opossum (*n* = 2)	—	0/4 (0.00%)	—	0/4 (0.00%)
		Raccoon (*n* = 4)	—	0/14 (0.00%)	—	0/14 (0.00%)
		Eastern Chipmunk (*n* = 1)	—	0/1 (0.00%)	—	0/1 (0.00%)
	July	Virginia Opossum (*n* = 3)	0/10 (0.00%)	0/13 (0.00%)	0/8 (0.00%)	0/31 (0.00%)
		Raccoon (*n* = 8)	0/20 (0.00%)	0/47 (0.00%)	—	0/67 (0.00%)
	August	Virginia Opossum (*n* = 3)	—	1/15 (6.66%)	0/1 (0.00%)	1/16 (6.25%)
		Raccoon (*n* = 4)	0/30 (0.00%)	3/14 (21.42%)	0/1 (0.00%)	3/45 (6.66%)
	September	Cat (*n* = 1)	0/1 (0.00%)	1/4 (25.00%)	—	1/5 (20.00%)
2021	July	Environment	—	0/6 (0.00%)	0/1 (0.00%)	0/7 (0.00%)
2022	April	Cattle (*n* = 13)	—	15/39 (38.46%)	0/7 (0.00%)	15/46 (32.60%)
Total			0/82 (0.00%)	20/222 (9.00%)	0/44 (0.00%)	20/348 (5.74%)

Mammalian hosts included cattle (*Bos taurus*), domestic cats (*Felis catus*), raccoons (*Procyon lotor*), Virginia opossum (*Didelphis virginiana*), and eastern chipmunk (*Tamias striatus*). Positive sampling events are grayed.

## Discussion

We report a first case study investigating the host ecology and potential introduction of *T. orientalis* Ikeda at a cow-calf farm, which includes ticks from the environment and hosts over multiple years. This study happened because of continued communication with the producer and the producer’s willingness to let us work on their farm; notably, we were able to learn how their farm likely became *T. orientalis* Ikeda positive because of their continued communication with us. *H. longicornis*, collected from this farm, are the first ticks detected with *T. orientalis* Ikeda in Tennessee based on retrospective screening. Importantly, this study highlights the significance of on-farm sampling and surveillance and reinforces recommendations associated with biosecurity using One Health concepts (19, 20).

Our studies began with opportunistic environmental and cattle sampling to detect *H. longicornis* populations and progressed to the assessment of other animals on the farm to determine if they also supported *H. longicornis* populations. No environmentally collected ticks were found to be pathogen positive on this farm, but these results could be due to inconsistencies in field collections between years in different habitat types. When we began sampling at this farm in 2020, producers attempted to manage their tick populations by changing from an open (cattle movement on and off the farm) to a closed herd (no cattle movement into the farm), managing pastures, and treating animals with on-host acaricides. While these actions reduced the tick population by 68% (10), cattle on this farm were confirmed as *T. orientalis* Ikeda positive 3 years after efforts began.

The one feline present on the farm was found with a *T. orientalis* Ikeda-positive *H. longicornis* tick. Companion animals, such as dogs and cats, are known to be preferred hosts for *H. longicornis* (9). While we were unable to sample blood from this host and screen it for pathogens, we did collect many engorged ticks from this cat, and only 1 was *T. orientalis* Ikeda positive (10 larvae and 4 nymphs were sampled). So, it is unlikely that the host was positive. Companion animals found to support pathogen-positive ticks are concerning because of their close association with human-influenced environments. Additionally, free-roaming companion animals have large home ranges and, due to their close association with humans, may also be moved long distances with them during travel, thus potentially increasing the dispersal of ticks and their associated pathogens.

Wildlife hosts were not *T. orientalis* Ikeda positive and unlikely to be an amplified host on this farm, but they were likely responsible for supporting and distributing immature populations of *H. longicornis* (7). Tick species, *Amblyomma americanum*, *Dermacentor* var*iabilis*, *Ixodes scapularis*, *I. cookei*, and *I. texanus*, were also found interacting with *H. longicornis* on wildlife, which is described as Farm 3 by (7). Although the only tick species screened for *T. orientalis* Ikeda was *H. longicornis*, additional studies need to be completed to investigate the role of other established tick species in the US in transmitting this pathogen. Readily available and abundant populations of wildlife that can sustain *H. longicornis* ticks in farm settings could potentially lead to transmission of positive ticks to infect cattle. Raccoons and Virginia opossums have large home ranges (21) and may have introduced ticks to this farm. Of note, this may be one of the first studies to confirm *T. orientalis* Ikeda-positive ticks on Ikeda-negative wildlife in the US.

Previously, Butler and Trout Fryxell (10) found that farmer-led integrated pest management strategies, which included cultural, chemical, and mechanical control, effectively reduced the number of *H. longicornis* ticks in the environment. It was later concluded that managing tick populations in the environment also reduced the number of ticks present on wildlife hosts (7). Promoting on-farm management strategies such as monthly bush-hogging, on-animal acaricides, and weekly environmental dragging could reduce tick abundance for farms experiencing *H. longicornis* infestations. Because wildlife species, such as raccoons, opossums, and white-tailed deer, can support large populations of *H. longicornis*, on-farm management strategies targeting these species with acaricide treatments or culling could be important for reducing large numbers of ticks (10, 22). Chemical applications should be applied in the warmer months when ticks are most abundant on wildlife, and the use of products with less of a residue concern, such as pyrethrins, could be used for animals intended for consumption (10, 23).

While we provided the producer with animal health and biosecurity information related to managing and reducing ticks and tick-borne diseases on cattle, ultimately, all producers make their own decisions. For example, the producers of this farm acknowledged that to save time and money, they reused syringes among cattle during vaccination. This behavior is a known risk to spreading tick-borne pathogens, as well as other pathogens, within herds, and this decision may have led to additional *T. orientalis* Ikeda-positive animals (24). Of note, we were not able to obtain blood from cattle, so the true prevalence of *T. orientalis* Ikeda on the farm is unknown; however, we were notified that some animals presented with theileriosis*-*like clinical signs. Keeping a closed herd reduced the chances of accidental introduction of a positive animal into their herd and onto their farm; however, neighboring farms kept open herds, and wildlife from those farms may have introduced *T. orientalis* Ikeda-positive ticks. Biosecurity methods, which are vital for disease control and prevention, are an important component to consider for reducing the spread of invasive ticks and associated pathogens (25). Biosecurity methods, such as quarantining, traffic control, acaricide application, hygiene, reproduction strategies, isolation, and vaccination, can be used to reduce pathogen and arthropod spread in farm settings (26–29).

We also confirmed that all blood samples from wildlife hosts were *Theileria* negative. Although farmers did not permit us the ability, future studies should promote blood collections from companion, wildlife, and livestock hosts on farms infested with *H. longicornis*. Understanding the mechanisms by which hosts distribute *T. orientalis* Ikeda could provide useful insights into reducing its prevalence in farm settings. Additionally, pairing PCR and serological testing for collected host blood samples would provide a better insight into past and present *T. orientalis* Ikeda infections. Finally, prospective studies should examine the use of management strategies for controlling *H. longicornis* and *T. orientalis* Ikeda by targeting wildlife that supports large populations of these tick species.

## Conclusion

This on-farm case study identified raccoons, Virginia opossums, and one domestic cat supporting *T. orientalis* Ikeda-positive *H. longicornis* ticks. Future studies should investigate targeting hosts that support *H. longicornis* populations with acaricide, as recommended by the product label, using techniques such as the 4-poster device, to investigate reducing the likelihood that a farm will become *T. orientalis* Ikeda positive over time. Integrated pest management techniques for *H. longicornis*, with the use of chemical on-animal, mechanical, and cultural controls, have been shown to reduce tick populations in the environment over time. Additionally, maintaining biosecurity techniques, such as changing gloves and not reusing syringes among animals, could be vital in ensuring that *T. orientalis* Ikeda pathogens are not being transmitted to naive hosts by mechanical inoculation. Finally, after engagement with producers, behaviors such as keeping an open herd, reusing needles, and yearly cultural control likely contributed to increased populations of *H. longicornis* and widespread transmission of *T. orientalis* Ikeda to cattle throughout the farm. Educating producers about the consequences associated with the establishment of *H. longicornis* and *T. orientalis* Ikeda on farms and teaching methods to reduce exposure, are vital to reducing transmission.

## Data Availability

The data presented in the study are deposited in the GenBank repository, accession number PX933207.
